# The Role of Formononetin in Osteoblast Function and Mineralization Potential with Deproteinized Bovine Bone Material

**DOI:** 10.3390/cimb46120851

**Published:** 2024-12-17

**Authors:** Ebru Haciosmanoglu Aldogan, Deniz Başaran, Bilgin Öner, Başak Günçer

**Affiliations:** 1 Department of Biophysics, Cerrahpasa Faculty of Medicine, Istanbul University-Cerrahpasa, 34098 Fatih, Istanbul, Turkey; 2 Department of Oral and Maxillofacial Surgery, Faculty of Dentistry, Istanbul University, 34093 Fatih, Istanbul, Turkey; dentiz@gmail.com (D.B.);; 3 Department of Biophysics, Istanbul Faculty of Medicine, Istanbul University, 34093 Fatih, Istanbul, Turkey; basak.varol@istanbul.edu.tr

**Keywords:** formononetin, osteoblasts, phytoestrogen, bone formation

## Abstract

Objectives: Dental bone formation involves various cellular and molecular mechanisms, and phytoestrogens such as formononetin (FORM) are promising because of their estrogenic, anti-inflammatory, and antioxidant effects. This study investigated the effect of FORM on osteoblast proliferation, differentiation, and mineralization in combination with spongiosa granulates (BO) in vitro. Materials and Methods: Human fetal osteoblast cells (hFOB1.19) were treated with increasing concentrations of FORM (1, 10, and 100 µg/mL), BO, or their combination. Cell proliferation was assessed using a MTT assay. Alkaline phosphatase (ALP) activity, intracellular Ca^2+^, and Pi levels were measured using ELISA. Vascular endothelial growth factor (VEGF) and osteocalcin expression levels were analyzed by western blotting. Results: Cell proliferation increased with FORM, with or without BO, after 6 days (*p* < 0.001). FORM and BO had a synergistic effect on ALP activity (*p* < 0.001). Intracellular Ca^2+^ and Pi levels were highest in the BO-FORM group, suggesting superior mineralization (*p* < 0.05). VEGF and osteocalcin expression was significantly upregulated with FORM, alone and with BO (*p* < 0.05), indicating improved angiogenesis and bone maturation over 9 days. Conclusions: FORM enhances osteoblast proliferation, differentiation, and mineralization potential, particularly in BO spongiosa granulates. These data support the in vitro potential of formononetin-phytoestrogen in promoting osteoblast differentiation and mineralization potential with BO. These findings suggest that FORM, combined with BO, could improve bone augmentation in clinical applications such as maxillofacial surgery. FORM shows valuable potential for clinical applications, such as maxillofacial surgery, by promoting faster and more effective healing.

## 1. Introduction

The treatment of bone defects caused by trauma, infection, cyst, jaw fractures, congenital anomalies, and atrophy of the maxillofacial region is among the main problems faced by maxillofacial surgeons, plastic and reconstructive surgeons, and orthopedists working in this field. In the treatment of these defects, autogenous grafts, xenogenic grafts, allografts, and alloplastic grafts are used depending on the defect type. Autogenous grafts obtained from a person’s bone do not cause foreign body reactions and are considered to be the gold standard. However, disadvantages such as the need for a second operation to obtain autogenous bone, risk of additional bleeding or infection in this area, and limited amount of bone tissue in the treatment of large defects have led researchers to search for alternative materials [1,2]. These limitations of autogenous grafts are further compounded by unfavorable outcomes, such as resorption observed during augmentation for implant placement. Factors such as limited availability, complexity of harvesting procedures, donor-site morbidity, and unpredictable nature of graft resorption underscore the challenges associated with their use [3,4].

The xenogeneic Bio-Oss^®^ spongiosa (BO, Geistlich Biomaterials, Wolhusen, Switzerland), consisting solely of the mineral component of bovine bone, was selected as the control bone substitute material due to its extensive documentation in the literature [5]. BO is available in both block and granular forms, with various particle sizes. These materials contain a demineralized bovine bone matrix that is free of organic remnants such as cells and extracellular components like collagen [6]. BO spongiosa granules are extensively utilized in maxillofacial regenerative procedures because of their osteoconductive properties and structural similarity to the human bone. Their porous architecture facilitates cell migration and vascularization, which are essential for effective bone regeneration. Studies have demonstrated their efficacy in sinus floor elevation and alveolar ridge preservation, making them standard the clinical practice. For instance, a randomized clinical trial compared Bio-Oss^®^ with Cerabone^®^ in maxillary sinus lifting, highlighting its effectiveness in promoting new bone formation [7]. Additionally, studies have evaluated the application of xenograft materials such as BO for sinus floor elevation, highlighting their effectiveness and clinical relevance [8].

Dental osteoblast differentiation and mineralization potential is a dynamic and tightly regulated process involving the activity of various cell types, primarily osteoblasts, which are responsible for bone matrix synthesis. This process is crucial to maintain the integrity and functionality of the jawbone and teeth. Several molecular markers, including alkaline phosphatase (ALP), osteocalcin, and vascular endothelial growth factor (VEGF), play critical roles in bone formation and regeneration by promoting osteoblast differentiation, matrix maturation, mineralization, and angiogenesis, making them essential indicators for evaluating bone metabolism in regenerative studies [9]. Alkaline phosphatase (ALP), a key enzyme produced by osteoblasts, plays a crucial role in early osteogenic differentiation and bone mineralization. Studies have consistently demonstrated that elevated ALP activity is indicative of active osteoblast function, and its measurement serves as a valuable indicator of the initiation of bone formation [10]. Vascular endothelial growth factor (VEGF) is a critical regulator of angiogenesis and formation of new blood vessels. VEGF stimulates endothelial cell proliferation and migration, contributing to the establishment of a vascular network within the bone [11]. Osteocalcin, a non-collagenous protein secreted by osteoblasts, is involved in the late stages of bone formation and contributes to matrix maturation and mineralization [12].

Osteoblasts are responsible for bone formation, and many hormones secreted by the body are involved in bone formation. Osteoporosis, which is observed in parallel with the loss of estrogen in the postmenopausal period, may negatively affect the healing of bone defects. Herbal estrogens, whose chemical structures are similar to those of estrogen, have been presented as an option for osteoporosis treatment. Herbal estrogens, also known as phytoestrogens, have been shown to have an anabolic effect on bone metabolism in various studies and have been the subject of many studies in recent years [13,14,15]. Formononetin (FORM) is a natural isoflavone found in various plants with diverse pharmacological properties, including its potential role in bone formation and maintenance [16]. FORM affects osteoblasts and signaling pathways and exhibits potential estrogenic effects by binding to estrogen receptors, thereby influencing bone density [17]. Given the well-established role of estrogen in maintaining bone health, the estrogenic activity of FORM is particularly significant. FORM has been shown to promote osteoblast differentiation and mineralization, underscoring its potential as a key player in bone health [18].

Although there are studies on the systemic effects of FORM as a phytoestrogen, on the healing of bone defects and wounds, there is no particular research on the effect of FORM combined with grafts in maxillofacial surgery [19,20]. Despite the well-documented positive effects of formononetin on bone and soft tissue metabolism, its potential to enhance the healing process and improve the quality of newly formed bone in combination with commonly used graft materials remains unclear. Based on the challenges and opportunities of treatment with grafts in maxillofacial surgery, this study aimed to evaluate the potential effects of FORM combined with graft materials in vitro. This study utilized Bio-Oss spongiosa granulates because of their frequent application in dental and maxillofacial surgeries as a reliable bone graft material. Specifically, we investigated whether FORM enhances osteoblast proliferation, differentiation, and mineralization potential in the presence of spongiosa granulates in vitro. By examining key indicators such as alkaline phosphatase (ALP) activity, intracellular calcium (Ca^2+^), and phosphate (Pi) levels, and the expression of osteocalcin and vascular endothelial growth factor (VEGF), this study explored the potential of FORM to enhance bone quality and accelerate healing in pathological or iatrogenic fractures treated with grafts in maxillofacial surgery. While these findings are promising, further research is needed to explain FORM’s efficacy and long-term benefits of FORM in clinical settings.

## 2. Materials and Methods

### 2.1. Chemicals, Reagents, and Dental Materials

Spongiosa granulates that deprotenized bovine bone material (DBBM, Geistlich Bio-Oss^®^, BO) (size is 1–2 mm) were acquired from Geistlich Pharma, Wolhusen, Switzerland. The FORM molecule (C_16_H_12_O_4_) used in our study was 98.0% (HPLC, T) and was obtained from TCI America. The human-derived osteoblast (hFOB 1.19) cell line was obtained from the American Type Culture Collection (CRL-11372, ATCC, Manassas, VA, USA).

### 2.2. Cell Culture and Treatment

Human fetal osteoblast cells (hFOB 1.19), an immortalized human-derived continuous osteoblast cell line, are widely used in osteogenic studies owing to their reproducibility and ability to provide consistent experimental results. For this study, the cells were cultured in DMEM-F12 (without phenol red, Life Technologies, Carlsbad, CA, USA) supplemented with L-glutamine, 10% fetal bovine serum, and 1% penicillin/streptomycin (Life Technologies) at 37 °C and 5% CO_2_. Cells were seeded in a 96-well culture plate (1 × 10^4^ cells/well) for the cell proliferation assay, and enzyme linked immunosorbent assay (ELISA), in a 6-well culture plate (1 × 10^6^ cells/well) for western blotting. After overnight incubation, the cells were treated with or without BO (3 mg/per well) once and FORM (1, 10, and 100 µg/mL) for 1, 6, and 9 d.

BO granules (3 mg/per well) were added to the culture wells at the beginning of the experiment and remained in the culture throughout the entire experimental period without removal or replacement. The stability and structural integrity of the BO granules under the culture conditions (37 °C, 5% CO₂) were confirmed through multiple approaches. Visual inspections during regular media changes, weight measurements before and after the experiment using a precision analytical balance, and microscopic examination all indicated that the granules retained their structure and composition without significant degradation. The deproteinized and mineralized nature of the BO granules, which makes them inherently resistant to breakdown, further ensured their continuous interaction with the osteoblasts. Media changes were performed carefully to avoid displacement or loss of the granules, maintaining consistent exposure to the cells. These findings collectively validate the stability of BO granules and their suitability for evaluating the synergistic effects of BO and FORM throughout the experimental timeline.

### 2.3. Cell Proliferation

Cell proliferation was evaluated on days 1, 6, and 9 using 1-(4,5-Dimethylthiazol-2-yl)-3,5-diphenylformazan (MMT, Sigma, St. Louis, MO, USA). After treatment with or without BO or FORM, MTT (5 mg/mL) was added to each well and incubated in the dark for 3 h at 37 °C. The supernatant was decanted, and DMSO was added to the cells and incubated for 30 min. The optical density of the supernatant was measured at 570 nm using a microplate reader (Epoch Microplate Spectrophotometer, Biotek, Winooski, VT, USA). Each experiment was repeated at least three times [21].

### 2.4. Alkaline Phosphatase (ALP) ELISA Assay

The ALP enzyme-linked immunosorbent assay (ELISA) was performed according to the manufacturer’s instructions to detect ALP expression in osteoblasts. The cells were treated with BO and FORM for 1, 6, and 9 days. The concentration of ALP in the cells was measured using an ALP ELISA Kit (Elabscience, Houston, TX, USA) with a microplate reader (BioTek) at 570 nm. Three parallel wells were used for each group [22].

### 2.5. Calcium and Phosphate Quantification Assays

To evaluate bone mineralization in vitro, intracellular calcium (Ca^2+^) and phosphate (P_i_) quantification assays were performed separately. Osteoblasts were seeded in 96-well plates and incubated with the FORM and BO-FORM combinations for 1, 6, and 9 days. The cells were collected and lysed using radioimmunoprecipitation assay (RIPA) buffer (Thermo Fisher Scientific, Waltham, MA, USA). The Ca^2+^ concentration in the cell lysates was determined using a colorimetric assay according to the manufacturer’s instructions (Sigma, MAK022, Kawasaki, Japan). The absorbance of samples was measured at 575 nm [23,24]. To measure free inorganic phosphate levels in the cells, a phosphate colorimetric assay was performed according to the manufacturer’s instructions (Sigma, MAK030). The absorbance was measured at 650 nm using a plate reader [25]. The intracellular Ca^2+^ and P_i_ concentrations were calculated using the formula: C = Sa/Sv, where C represents the calcium concentration in the sample, Sa is the amount of calcium in the unknown sample (μg) determined from the standard curve, and Sv is the sample volume (μL). The samples were analyzed in duplicate.

### 2.6. SDS-PAGE and Western Blot Analysis

Osteoblasts were harvested on day 9 and lysed using RIPA lysis buffer (Thermo Fisher Scientific). The protein concentration was measured using a Qubit protein assay kit (Thermo Fisher Scientific), and protein samples (50 µg) were denatured with 5× loading buffer at 95 °C for 5 min. Following separation by 12% SDS-PAGE, proteins were transferred to nitrocellulose membranes [26]. Membranes were blocked with 5% BSA in Tris-buffered saline-Tween (TBST) to prevent nonspecific binding. Membranes were incubated overnight with primary antibodies against osteocalcin (1:500, Abcam, Cambridge, UK), Vascular Endothelial Growth Factor (VGEF, 1:500, Enzo Life Sciences, Long Island, NY, USA) and β-actin (1:1000, Cell Signaling, Danvers, MA, USA) followed by alkaline phosphatase-conjugated secondary anti-rabbit antibody (1:5000, Abcam, Cambridge, UK) to detect the proteins of interest using enhanced chromogenic antibodies (Thermo Fisher Scientific). Membranes were washed with TBST at the end of each step. Immunoreactive bands were visualized using a colorimetric detection kit (NBT-BCIP; Thermo Fisher Scientific), and protein bands were analyzed using ImageJ 1.46 r (NIH, Bethesda, MD, USA) [22].

### 2.7. Statistical Analysis

Data are expressed as mean ± SEM. Data were compared using two-way ANOVA using GraphPad Prism 6 (San Diego, CA, USA). Differences were considered statistically significant at *p* < 0.05.

## 3. Results

### 3.1. Effects of FORM on Cell Proliferation

**Figure 1:**
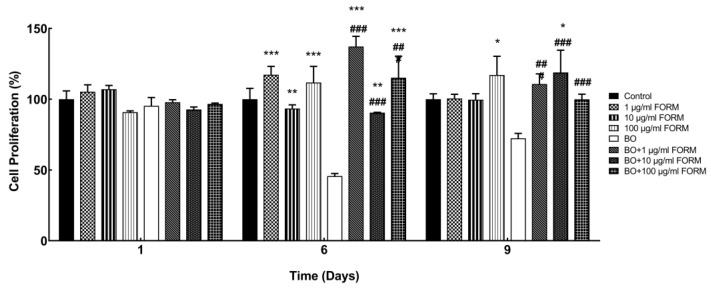
Cell proliferation of osteoblasts on days 1, 6, and 9. The control (0 μM) was set at 100%. Each sample was measured in triplicates. Values represent the mean ± SEM of three independent experiments. * *p* < 0.05, ** *p* < 0.01, *** *p* < 0.001 compared with the control; ^#^ *p* < 0.05, ^##^ *p* < 0.01 ^###^ *p* < 0.001 compared with BO.

The proliferation of osteoblasts treated with spongiosa granulates (BO) and formononetin (FORM) was assessed on days 1, 6, and 9 by the MTT assay. FORM and BO + FORM treatments significantly increased osteoblast cell proliferation after 6 days in the control and BO groups. However, cell proliferation decreased with only BO treatment, and treatment with BO and FORM significantly increased cell proliferation on days 6 and 9 (Figure 1). These findings suggest that both BO and FORM enhance osteoblast proliferation, with FORM demonstrating a more pronounced effect at higher concentrations over time.

### 3.2. Effects of FORM on ALP Activity

**Figure 2:**
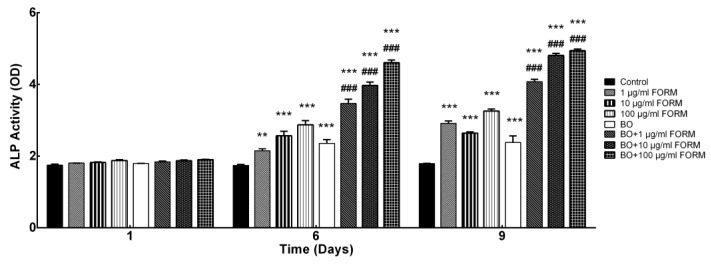
Effects of FORM on Alkaline phosphatase (ALP) activity on days 1, 6, and 9. Values are means ± SEM of three independent experiments. * *p* < 0.05, ** *p* < 0.01, *** *p* < 0.001 compared with the control; ^#^ *p* < 0.05, ^##^ *p* < 0.01, ^###^ *p* < 0.001 compared with BO.

ALP activity is a marker of osteoblast differentiation in the early stage [27] and was measured on days 1, 6, and 9 to understand the contribution of FORM with and without BO to osteoblast differentiation and bone mineralization. No significant changes in ALP activity were observed on day 1 after treatment with BO or FORM. However, on days 6 and 9, BO and FORM significantly induced ALP activity, both separately and in combination. BO and FORM treatments resulted in significant increases (*p* < 0.005 and *p* < 0.001, respectively). FORM enhanced ALP activity in the osteoblast cell line alone, compared to the control and BO alone groups, and showed a significant synergistic effect with BO treatment (*p* < 0.001) (Figure 2).

These results indicate that FORM, particularly at higher concentrations, significantly enhanced ALP activity over time, suggesting its potential role in promoting osteogenic differentiation and bone formation. In addition, increased ALP activity, as observed with FORM with BO, indicates a better bone-regeneration capacity of this graft material with FORM.

### 3.3. Effect of FORM on Intracellular Calcium and Phosphate Concentration

**Figure 3:**
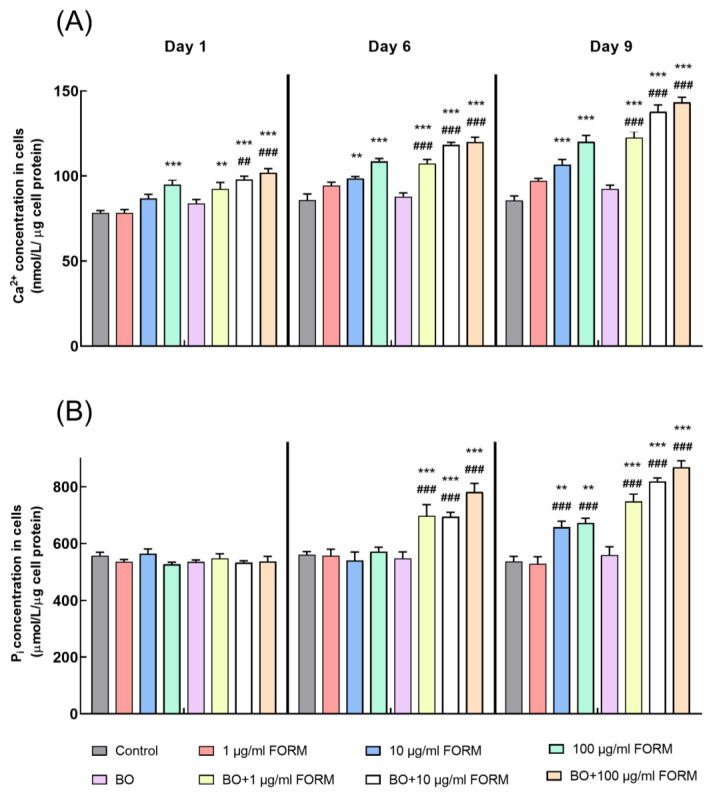
Intracellular calcium (Ca^2+^) (**A**) and phosphate (Pi) (**B**) levels in osteoblasts treated with FORM and BO+FORM. Values are means ± SEM of three independent experiments. * *p* < 0.05, ** *p* < 0.01, *** *p* < 0.001 compared with the control; ^#^ *p* < 0.05, ^##^ *p* < 0.01, ^###^ *p* < 0.001 compared with BO.

This study evaluated the intracellular calcium (Ca^2+^) and phosphate (Pi) levels to assess bone mineralization in vitro. Osteoblasts were treated with varying concentrations of FORM and BO-FORM s for 1, 6, and 9 days, and the intracellular concentrations of Ca^2+^ and Pi were measured. Treatment with FORM (1 μg/mL) resulted in a moderate increase in Ca^2+^ levels compared to the control, whereas higher concentrations (10 μg/mL and 100 μg/mL) of FORM resulted in significantly higher calcium levels on day 1. Similar to the 1-day incubation, on days 6 and 9, the BO-FORM combination treatment significantly elevated intracellular Ca^2+^ concentrations (*p* < 0.005 and *p* < 0.001, respectively) (Figure 3A). Notably, 100 μg/mL BO-FORM combination led to the highest calcium uptake, indicating a synergistic effect of BO and FORM in promoting calcium deposition in osteoblasts.

The changes in Pi levels showed a slight increase with 1 μg/mL FORM treatment, with more substantial increases observed at 10 and 100 μg/mL FORM concentrations on day 1. Pi concentration increased further in all treatment groups, with the BO-FORM combination showing the highest levels on day 6. The 100 μg/mL BO-FORM combination demonstrated the most significant (*p* < 0.001) Pi increment, suggesting enhanced mineralization activity (Figure 3B).

The data indicated that both calcium and phosphate uptake were significantly enhanced by the BO-FORM combination compared with FORM alone. The highest concentrations of BO-FORM consistently yielded the greatest mineralization effects, underscoring the potential of BO-FORM combinations to promote osteoblastic activity and bone mineralization.

### 3.4. Level of VEGF and Osteocalcin Expression

**Figure 4:**
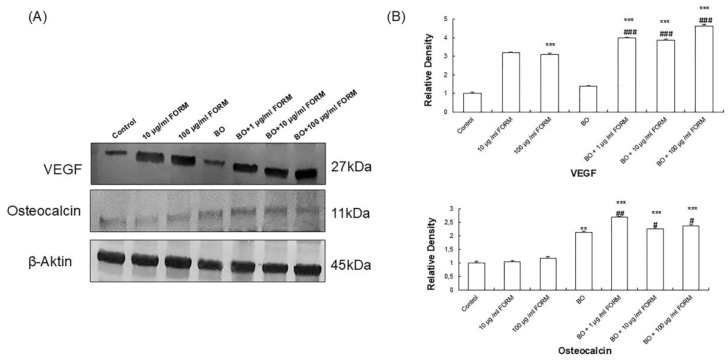
Effects of FORM on Vascular endothelial growth factor (VEGF) and osteocalcin expression after 9 days. (**A**) Western blot analysis. (**B**) Protein expression levels of VEGF and osteocalcin were analyzed using ImageJ software. Values are expressed as means ± SEM of three experiments. Values are mean ± SEM of three independent experiments. * *p* < 0.05, ** *p* < 0.01, *** *p* < 0.001 compared with the control; ^#^ *p* < 0.05, ^##^ *p* < 0.01, ^###^ *p* < 0.001 compared with BO.

To examine the effect of FORM on VEGF and osteocalcin levels, western blotting was performed with osteoblasts incubated for 9 days according to the cell proliferation and ALP activity results. Although VEGF expression was significantly increased with FORM treatment, and the synergetic effect of BO and FORM incubation occurred on VEGF levels similar to ALP activity, no significant changes were observed with BO treatment alone. (Figure 4A). While osteocalcin levels were induced by FORM and BO separately, treatment with 1 µg/mL FORM and BO together increased osteocalcin expression significantly compared to the control and BO alone groups (Figure 4B). These results demonstrate that FORM induced the expression of bone formation markers with BO, and these effects could be helpful in using FORM with BO for bone development.

## 4. Discussion

Bone augmentation procedures are frequently applied in maxillofacial surgery to restore old bone volume by repairing bone tissue losses that occur after traumatic tooth extraction, cyst, and tumor operations with appropriate graft materials. At the end of the healing process, the graft materials used in the augmentation procedures could not remain in the initial volume in which they were placed, and resorption was observed in the relevant area. In successful augmentation, it is expected that the mineralized tissue obtained after the healing period will have properties similar to the bone in the recipient area and will preserve the volume of the first application as much as possible. To achieve these expectations, the graft material must first be in a structure that allows cell migration from the recipient site into the graft and mechanically functions as a roof that can create the desired bone volume. After providing this structure, the first vascular structures are formed in the augmentation area, and new bone formation occurs with cellular activities. Thus, finding an agent that can contribute to anabolic effects in the tooth environment is important for the success of augmentation [28,29,30].

Estrogens play a crucial role in the proper development and maintenance of skeletal structure, contributing to bone mineral density, mass, and regulation of bone turnover rates. In addition, a balanced interaction between osteoclasts and osteoblasts is essential for successful dental implant outcomes [31]. Phytoestrogens are non-steroidal compounds derived from plants that interact with estrogen receptors (ERs) and exhibit estrogen-like effects. They can be categorized into three main classes: isoflavones, coumestans, and lignans. These compounds are known to contribute to the prevention and treatment of various health conditions, including cardiovascular diseases, osteoporosis, diabetes, obesity, menopausal symptoms, kidney diseases, and certain types of cancer [32]. One study compared the effectiveness of two forms of phytoestrogens in soybean as a graft material for parietal bone defects in New Zealand white rabbits. The results of this study showed an increase in bone formation after 14 days of treatment with both forms of phytoestrogens.

Formononetin (FORM) is phytoestrogen with osteogenic, angiogenic, and wound-healing activities [33]. Some phytoestrogens have been reported to contribute to bone metabolism by inhibiting osteoclastic activity [28,29,33]. Huh et al. (2014) reported that formononetin exerts an antiresorptive effect by preventing osteoclast activation [34]. In this study, FORM increased osteoblast proliferation, and it was thought that FORM could protect bone volume and reduce cell destruction during the bone-augmentation process. Kaczmarczyk-Sedlak et al. (2013) investigated how formononetin, applied for 4 weeks to rats with osteoporosis, affected the mechanical properties and chemical structure of the bone. It has been reported that formononetin significantly reduces the water content of the bone and slightly improves its mechanical properties. As a result of this study, formononetin, reported as a supplement, could be effective in preventing osteoporosis [35]. ALP activity in osteoblasts stimulated with insulin-like growth factor corresponds to osteogenesis in the first, sixth, and ninth days [36]. In this study, the possible early effect of formononetin on ALP activity was evaluated, as in Palermo’s study, which showed that ALP activity increased to the highest level on the 9th day. At the same time as Palermo’s study, it was revealed that ALP activity was increased to the highest level at the 9th day. FORM plays a role in the calcification process associated with BO. It has been shown that elevated concentrations of calcium and phosphate is crucial for in vitro mineralization in a rat osteoblast-like cell-culture system [37]. In this study, the increased intracellular calcium (Ca^2+^) and phosphate (Pi) levels observed with the FORM and BO-FORM treatments highlighted the role of FORM in promoting mineralization potential. Although an increase in intracellular calcium (Ca^2+^) contributes to bone mineralization, it is thought to contribute to the bone-healing process only indirectly. In an in vitro study, it was reported that formononetin could reduce protein synthesis in osteoarthritic cells and increase the protein synthesis of normal osteoblasts [38]. Osteogenic differentiation of mesenchymal stem cells obtained from rats in culture medium for 16 days in cell culture showed that osteocalcin is a reliable ossification marker before the occurrence of ossification [39]. In our study, the expression of osteocalcin was examined with and without FORM and BO, and FORM- induced osteocalcin levels were significantly increased with BO. Vascular endothelial growth factor (VEGF) plays an important role in soft tissue and bone healing. In addition, VEGF levels can affect ALP levels, bone formation, and mineralization in osteoblasts [11]. In the present study, FORM-induced VEGF expression was significantly higher with BO. This result revealed that FORM can greatly contribute to the formation of the first vascularization during bone healing. Although we obtained promising results using FORM with BO in the bone-healing process, this in vitro study has certain limitations. The use of a single osteoblast cell line (hFOB 1.19) limits the applicability of these findings to other cell types involved in bone formation. Additionally, the lack of in vivo validation means that the study cannot fully replicate the complex biological processes that occur in living systems. The long-term effects of FORM and BO on bone regeneration remain unclear. Future studies should focus on in vivo validation of FORM’s osteogenic effects to assess its performance under physiological conditions. Additionally, exploring the efficacy of FORM in combination with other biomaterials, such as allografts or synthetic scaffolds, could expand its clinical applications. Further in vivo studies are necessary to compare the local or systematic application of FORM, considering factors such as bioavailability, local concentration at the target site, and systemic side effects. Investigating the long-term outcomes and mechanisms underlying its bone-regenerative potential will further clarify its role in maxillofacial surgery.

## 5. Conclusions

In conclusion, FORM exhibits significant potential for enhancing osteoblast activity and mineralization potential, particularly when used in combination with BO spongiosa granules. The findings of this study provide a basis for further research on the clinical applications of FORM in bone grafting and maxillofacial surgery, with the goal of improving patient outcomes through enhanced bone regeneration. These results indicated that FORM has the potential to be used as a supportive drug for augmentation with graft materials. However, owing to the limitations of this in vitro research, further in vivo and clinical studies are essential to examine the efficacy of FORM on dental bone formation and healing processes.

## Data Availability

Data are available upon request from the corresponding author.
